# Supplementary figures and images for: Overexpression of Sugarcane ScDIR Genes Enhances Drought Tolerance in Nicotiana benthamiana
Source: Int J Mol Sci. 2022 May 10;23(10):5340. doi: 10.3390/ijms23105340 (PMC9141896; doi:10.3390/ijms23105340)

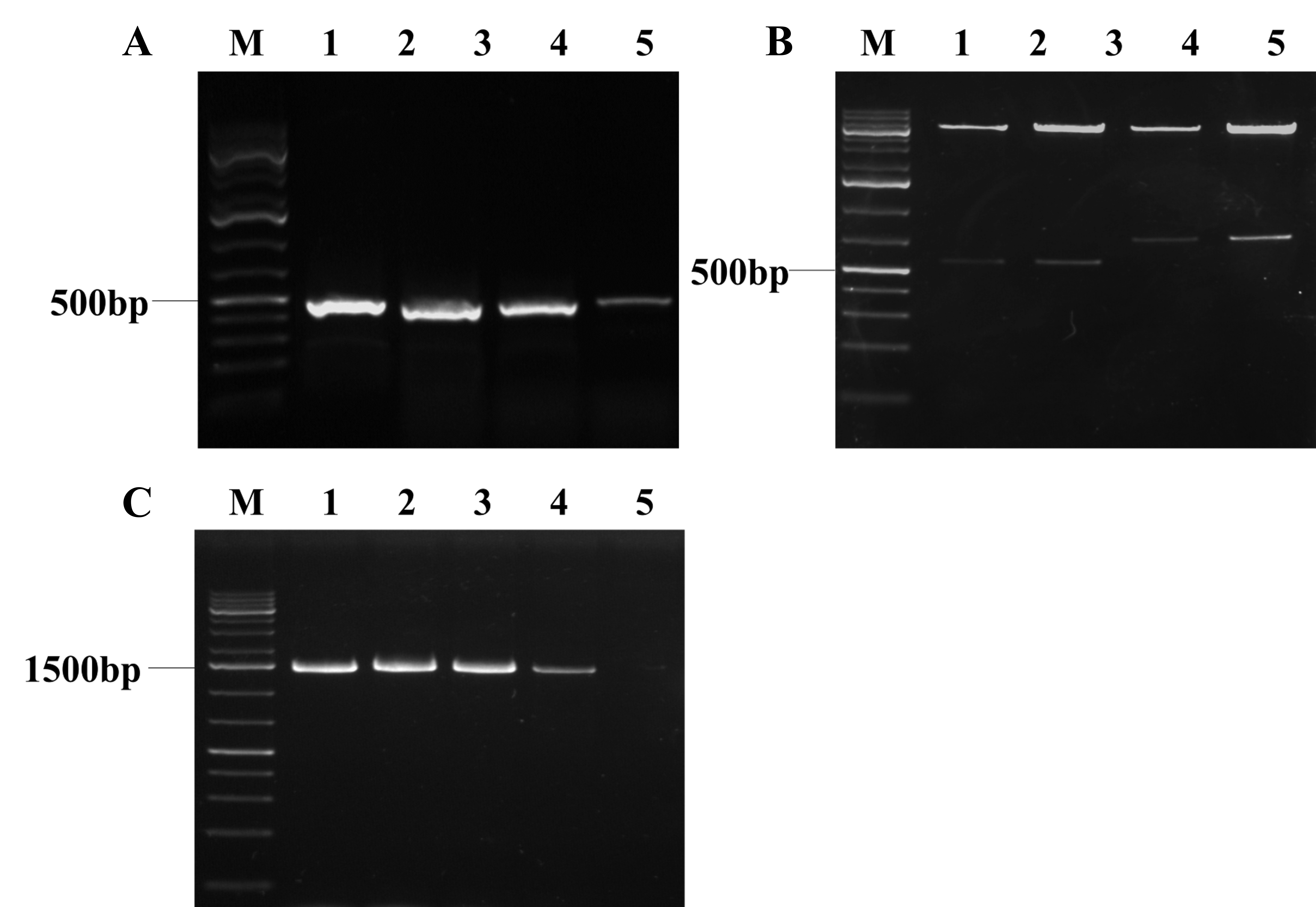

Supplement: Supplementary file 1 [file ijms-23-05340-s001.zip › Figure S1.tif]

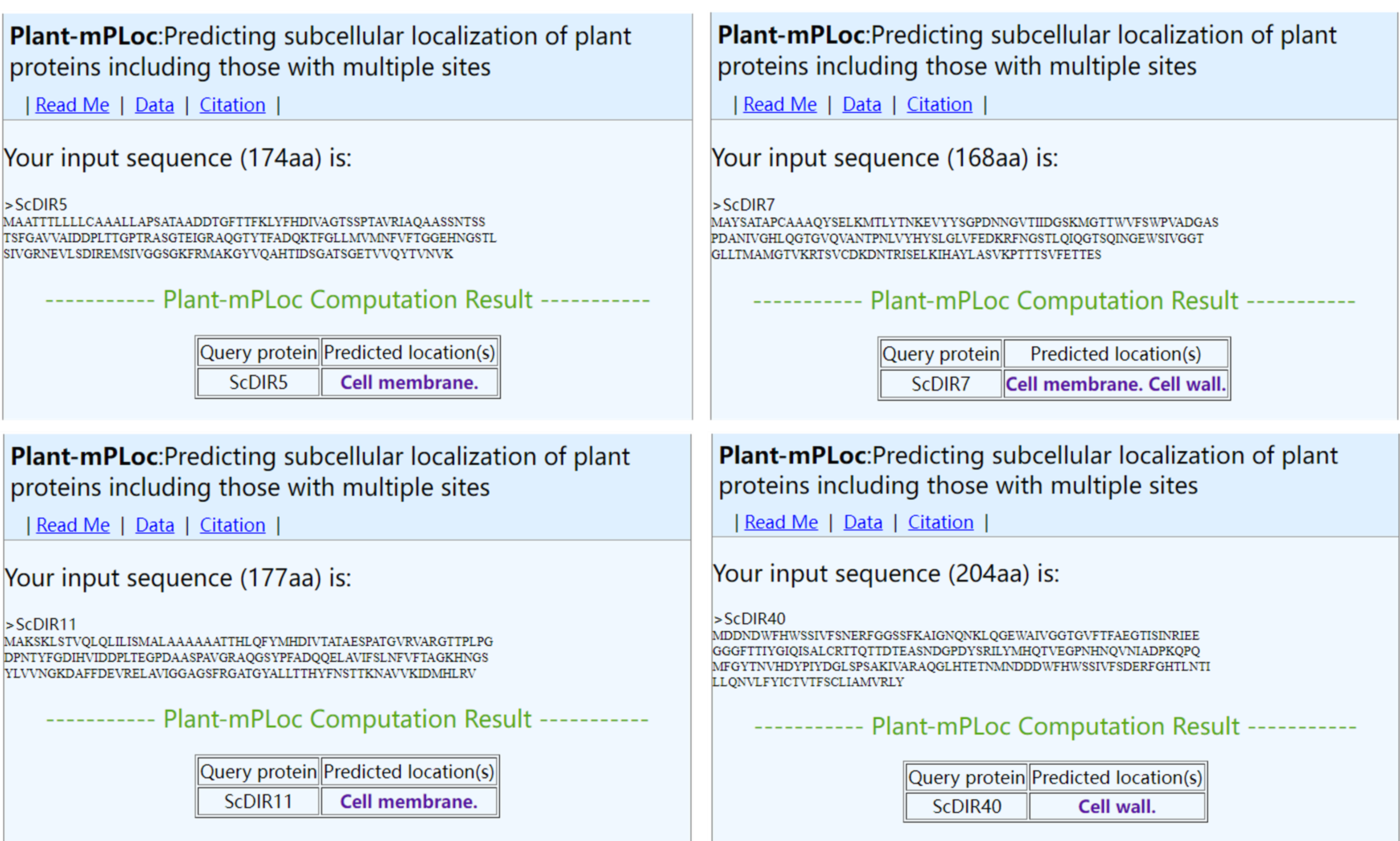

Supplement: Supplementary file 1 [file ijms-23-05340-s001.zip › Figure S2.tif]
